# Understanding the Molecular Aspects of Tetrahydrocannabinol and Cannabidiol as Antioxidants

**DOI:** 10.3390/molecules181012663

**Published:** 2013-10-14

**Authors:** Rosivaldo S. Borges, João Batista Jr., Rommel B. Viana, Ana C. Baetas, Ednilsom Orestes, Marcieni A. Andrade, Káthia M. Honório, Albérico B. F. da Silva

**Affiliations:** 1 Institute of Health Sciences, Federal University of Pará, Belém 66075-110, PA, Brazil; E-Mails: jbatistajr@hotmail.com (J.B.J.); crisbaetas@ufpa.br (A.C.B.); marcieni@ufpa.br (M.A.A.); 2 Institute of Chemistry of São Carlos, University of São Paulo, São Carlos 13560-970, SP, Brazil; E-Mails: rommelbv@yahoo.com.br (R.B.V.); eorestes@gmail.com (E.O.); alberico@iqsc.usp.br (A.B.F.S.); 3 School of Arts, Sciences and Humanities, University of São Paulo, São Carlos 03828-000, SP, Brazil; E-Mail: kamaho@gmail.com

**Keywords:** SAR, antioxidant, DFT, cannabinoids, CBD, electronic, THC

## Abstract

An antioxidant mechanism of tetrahydrocannabinol (THC) and cannabidiol (CBD) were compared with a simplified model of α-tocopherol, butylhydroxytoluene and hydroxytoluene in order to understand the antioxidant nature of THC and CBD molecules using DFT. The following electronic properties were evaluated: frontier orbitals nature, ionization potential, O-H bond dissociation energy (BDE_OH_), stabilization energy, and spin density distribution. An important factor that shows an influence in the antioxidant property of THC is the electron abstraction at the phenol position. Our data indicate that the decrease of the HOMO values and the highest ionization potential values are related to phenol, ether, and alkyl moieties. On the other hand, BDE_OH_ in molecules with the cyclohexenyl group at *ortho* position of phenol are formed from lower energies than the molecules with an ether group at the *meta* position. In the light of our results, the properties calculated here predict that THC has a sightly higher antioxidant potential than CBD.

## 1. Introduction

**Figure 1:**
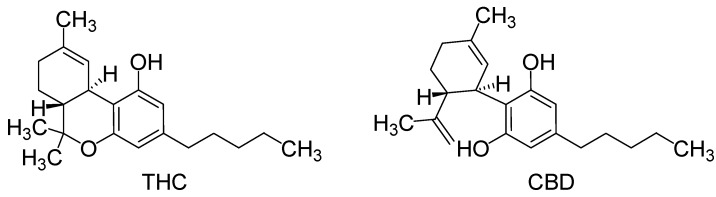
Structures of tetrahydrocannabinol (THC) and cannabidiol (CBD).

The discovery of endocannabinoids as pain modulators has opened new mechanistic perspectives [1]. The endogenous ligands of cannabinoid receptors CB1 and CB2, mainly metabolized by the fatty acid amide hydrolase and the monoacylglycerol lipase, induce antinociceptive effects [2,3]. Similarly, the activation of the lipase by exogenous ligands of cannabinoid receptors, particularly CB1, induces antinociception in various acute pain tests in rodents [2,4,5], but also in several animal models of chronic pain [6]. Moreover, the combination of tetrahydrocannabinol (THC) and cannabidiol (CBD) (Figure 1) is proposed in the treatment of pain for patients with multiple sclerosis [7].

THC, like other cannabinoids that contain a phenol group, possess mild antioxidant activity, sufficient to protect neurons against oxidative stress, such as that produced by glutamate-induced excitotoxicity [8]. In fact, tests in rats indicate that THC prevents hydroperoxide-induced oxidative damage as well as or better than other antioxidants in a chemical system and neuronal cultures induced by Fenton reactions [9]. Other work using mice showed that low doses of THC reduce the progression of atherosclerosis [10]. Thus, experimental evidence shows that THC can prevent Alzheimer’s disease in an animal model by preventing the inflammation caused by microglia cells which are activated by the binding of amyloid protein [11]. From *in vitro* experiments, THC at extremely high concentrations, which could not be reached with commonly-consumed doses, caused the inhibition of plaque formation, which is associated with Alzheimer’s disease, and this approach provides better results than the currently-approved drugs [12]. Further, THC may also be an effective anti-cancer treatment, since some studies show reduction in the tumor size in mice [13,14], as well as in humans with glioblastoma multiforme [15].

Currently, research has also shown that past claims of brain damage from cannabis use cannot be confirmed [16]. Instead, recent studies with synthetic cannabinoids show that the activation of CB1 receptors can facilitate the neurogenesis [17], as well as neuroprotection [18], and can even help the prevention of the natural neural degradation from neurodegenerative diseases. These findings, along with the researches on the CB2 receptor, have confirmed the medicinal use of cannabinoid-like substances [19,20]. THC is considered a CB1 and CB2 agonist [21] and its metabolism occurs mainly in the liver via oxidation reactions by cytochrome P450 enzymes [22].

In accordance to the literature, two main mechanisms are proposed to explain the protective role as antioxidant of phenol derivatives [22,23,24,25,26,27,28,29,30,31]: One is the H-atom transfer, in which a free radical R^•^ removes a hydrogen atom from the antioxidant (ArOH): Equation (1), and the other one is a one-electron transfer mechanism, where the antioxidant can donate an electron to the radical: Equations (2) and (3). In addition to these mechanisms, the radicals arising from both reactions (ArO• and ArOH•+) must be stable to prevent chain radical reactions.


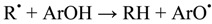
(1)


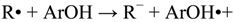
(2)


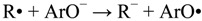
(3)

**Figure 2:**
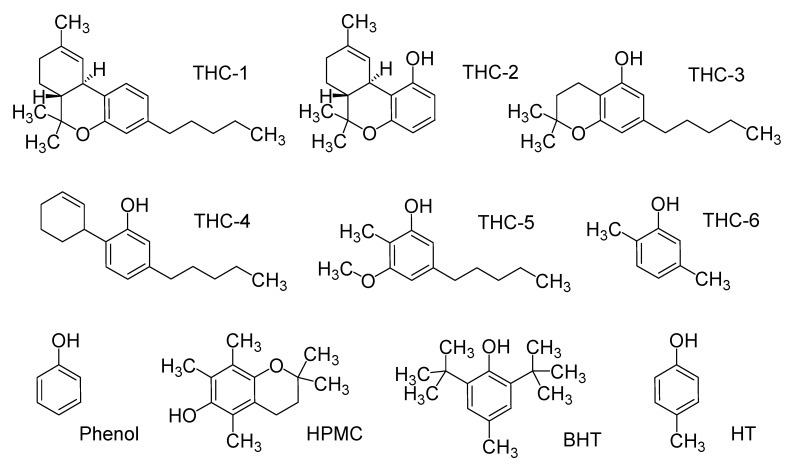
Structure of simplified model of tetrahydrocannabinol (THC) and classical antioxidants.

The main goal of the present study was to obtain a relationship between electronic properties and the antioxidant capacity of THC and CBD, generating a useful methodology to investigate their possible antioxidant mechanisms and the potential of these important substances. We are interested in understanding the role played by the different structural features of the THC molecule, defining how these functional groups are responsible for its antioxidant properties. Thus, we have undertaken a systematic study of the influence of the phenol, dimethylpyran, methylcyclohexene, and *n*-pentyl groups on the antioxidant properties of THC and CBD. In addition, the results from CBD and THC derivative properties were compared with a simplified model of α-tocopherol (HPMC, 6-hydroxy-2,2,5,7,8-pentamethylchroman), butylhydroxytoluene (BHT) and hydroxytoluene (HT), as can be seen in Figure 2, based on several comparative features among the antioxidant properties of these molecules [9,32]. In this study, some structural and electronic characteristics of THC and CBD such as ionization potential (IP), the energies of the highest occupied molecular orbital (HOMO) and the lowest unoccupied molecular orbital (LUMO), hydroxyl bond dissociation energy (BDE_OH_), and spin density distribution were obtained using the Density Functional Theory (DFT) methodology with the objective of shedding a light on the possible oxidation mechanism of THC and its relationship with the observed cytoprotective properties.

## 2. Results and Discussion

**Figure 3:**
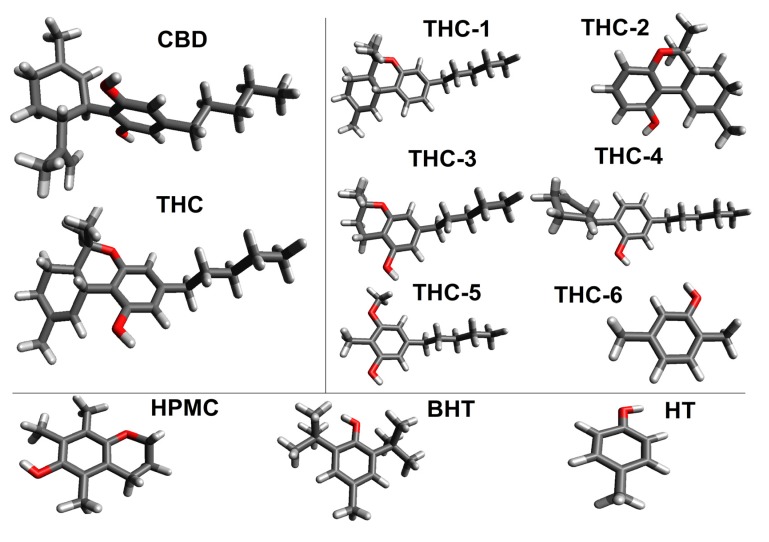
Optimized structures of the neutral molecules.

The final molecular geometry of THC and CBD was obtained with the B3LYP/6-311++G(d,p) methodology in an IEFPCM continuum solvent model and are displayed in Figure 3. From the optimized geometries, it is possible to observe that the structure of THC does not have intermolecular hydrogen bond from the oxygen atom of the phenol group. In fact, the hydrogen of the phenol moiety has a great electronic repulsion with the methylcyclohexene ring due to the difference of polarity.

In general, the three rings (phenol, pyran and cyclohexene) of THC are responsible for its characteristics as a rigid molecule. On the other hand, only the alkyl moiety has several conformations; while this group has a preferred *anti*-conformation among its methylene groups. The geometry of CBD is not planar, having stronger repulsion between both hydroxyl groups and the limonene ring.

**Figure 4:**
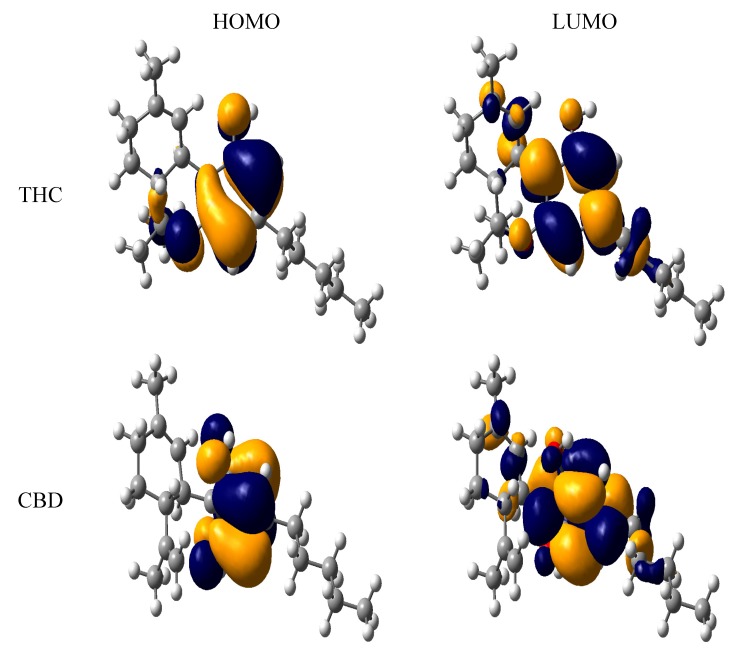
HOMO and LUMO of tetrahydrocannabinol (THC) and cannabidiol (CBD).

Table 1 presents the frontier orbital energies, ionization potential and the O-H bond dissociation energies of the molecules studied here. Comparing the HOMO results from THC and CBD with the HPMC one, a very similar value can be seen. The THC and CBD molecules showed HOMO values of −6.03 eV and −6.18 eV, respectively. This result indicates that the ether moiety decreases the HOMO values. In fact, the ether group has a great influence on the HOMO and LUMO contributions, as shown in Figure 4. The electronic effect in the phenolic compound is influenced by other functional groups that have a participation in the inductive or resonance effects and can to indicate its reactive point of scavenging free-radicals qualitatively because the H-abstraction after electron transfer. The number of resonance structures or electron donating groups linked at the *ortho*- or *para*-positions of the phenol moiety can be related to the highest nucleophilicity of THC than CBD.

**Table 1:** Frontier orbital energies (HOMO and LUMO, in eV), ionization potential (IP, in kcal mol^−1^) and the O-H bond dissociation (BDE, in kcal mol^−1^) of the molecules.

Molecules	HOMO	LUMO	IP	BDE
CBD	−6.18	−0.38	142.33	85.63
THC	−6.03	−0.36	138.88	84.49
THC-1	−6.04	−0.51	138.86	-
THC-2	−6.11	−0.44	140.23	85.11
THC-3	−6.03	−0.31	138.92	84.94
THC-4	−6.19	−0.51	142.13	84.86
THC-5	−6.16	−0.31	140.63	85.68
THC-6	−6.24	−0.52	142.06	84.81
Phenol	−6.29	−0.42	148.82	87.93
HPMC	−5.56	−0.39	125.77	76.01
BHT	−5.97	−0.36	136.71	78.48
HT	−6.25	−0.64	142.54	84.86

In fact, the HOMO energy is an important electronic parameter for describing the antioxidant ability of a molecule, since this property can be related to electron transfer reactions. A molecule with low values of HOMO energy has a weak electron donating ability. Otherwise, a higher HOMO energy implies that the molecule is a good electron-donor [33,34], while the LUMO energies did not have significant differences. It is interesting to note that THC derivatives had the highest value of HOMO energy, indicating their high electron donating ability. Besides, we also calculated the main atomic contributions for HOMO and LUMO, which are displayed in Figure 4. The most nucleophilic positions were determined by the main HOMO contributions of the phenol moiety. Additional contributions from the ether and alkyl groups linked to the phenol moiety can also be observed. The coplanarity between these groups and the phenol moiety is contributing to the nucleophilicity and the *n*-pentyl moiety has a little contribution for the nucleophilicity. Analyzing the LUMO contributions, we can see that the aromatic region is more electrophilic than the hydroxyl moiety. The same behavior is observed for the phenol group of the CBD compound.

The nucleophilicity of THC can also be expressed by the ionization potential value (IP), which is calculated as the necessary energy for the abstraction of an electron in the molecule. In fact, IP represents the easiness of the electron donation of THC due to the electron abstraction is the first antioxidant mechanism. Therefore, molecules with low IP values can more easily undergo oxidation. The results showed that THC has an IP value of 138.88 kcal mol^−1^, while CBD has an IP value of 142.33 kcal mol^−1^. This difference indicates that the substitution of the pyran and cyclohexenyl rings at the *ortho*- and *para*-positions of the phenol ring in the THC decreases its IP value and also increases its electron-donating capacity. The HPMC ionization potential energy in this study is 20 kcal mol^−1^ lower than the result predicted by Leopoldine *et al.* [23], which is due to the different level of theory applied in each study. In our case, after the optimization with B3LYP/6-311++G(d,p) method in a IEF-PCM water solvent model, we also calculated a single point calculation with M06-2X/6-311++G(3df,3pd) and as far as the comparison with the literature is concerned we predicted a better result than Leopoldine *et al.* [23], which is also valid for the BDE_OH_ energy. Comparing the IP results between HMPC with THC and CBD, it can be seen that HPMC can easily suffer oxidation. On the other hand, THC presents a similar IP value as BHT, which is in good agreement the experimental investigation of Hampson *et al.* [9], who demonstrated that THC and BHT present similar ability to prevent dihydrorhodamine oxidation. By cyclic voltametric experiments, Hampson *et al.* [9] also showed the similar ability of CBD. In addition, Hamelink *et al.* [32] observed similar antioxidant ability between CBD, HT and a-tocopherol with cyclic voltametric profiles.

The hydroxyl bond dissociation energies for the molecules represent the ease of hydrogen donation presented by the THC derivatives to give semiquinone derivatives and the influence of the alkyl moiety on the THC structure, which indicates that molecules with a low BDE_OH_ have more important substituents for the antioxidant activity. The BDE_OH_ value for THC is of 84.49 kcal mol^−1^. Molecules with cyclohexene in the *ortho* position had more influence on the BDE_OH_ values, such as the derivatives with the ether moiety in the *meta* position.

These results show a good relation with the HOMO and IP values. Our results show that the antioxidant capacity for this compound (THC) can be mainly determined by the stability of the semiquinone radical, generated after the hydrogen abstraction. The BDE_OH_ energies in molecules with the cyclohexenyl group in the *ortho* position of the phenol are formed with less energy than in molecules with the ether moiety in the *meta* position. The highest energy found for the hydrogen abstraction in *ortho*- and *meta*-positions is facilitated due to the π-delocalized system between the cyclohexene and pyran rings, respectively. The increase of the BDE_OH_ values for CBD (85.63 kcal mol^−1^) is related to a loss of the planar conformation between the phenol, pyran and cyclohexene rings. In addition, the semiquinone form has an interaction with alkene groups of the limonene ring.

**Figure 5:**
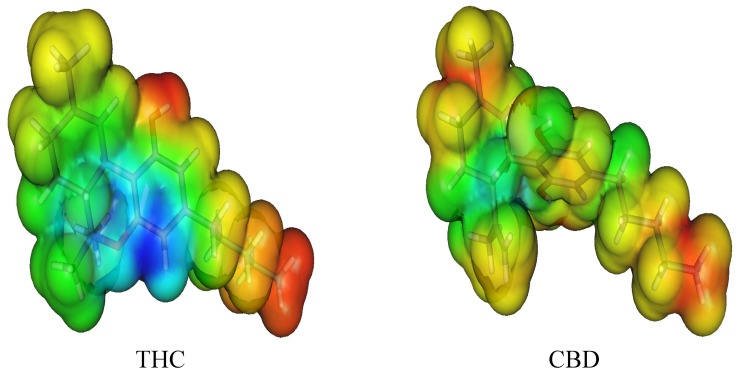
Molecular electrostatic potential (MEP) surface of cannabinoid derivatives.

To assess this behavior, we decided to obtain the Molecular Electrostatic Potential (MEP) surfaces for THC and CBD, as can be seen in Figure 5. All oxygen moieties showed a negative potential (red) placed at the phenol and ether groups, being the positive potential (blue) located at all hydrogen atoms, mainly located in the phenol moiety. The electrostatic proﬁle of THC is signiﬁcantly different when compared to CBD. This fact highlights the two fused-rings directly attached to the phenol moiety as responsible for this electronic behavior. However, in THC, the antioxidant activity can be mainly determined by the stability of the cation free-radical and these reactive radical species are generated after the electron abstraction during the oxidation process. Thus, the electronic effects, such as the inductive effect of the alkyl moiety, and the resonance effects, due to the ether group, are mainly responsible for the cation free-radical stabilization. In fact, the hydroxyl moiety of THC is more reactive than the hydroxyl group of the other phenol molecules studied here due to the resonance effect and its cation free-radicals are formed with minor energy. Therefore, the compounds with more resonance structures are more stable and present the lowest IP value.

These results are important as the oxidative stress can be inhibited by the presence of various antioxidants, which can act on different processes [23]. The chain-breaking antioxidants are one of the antioxidant types, for example THC, which inhibits the process of oxidative stress by scavenging the free-radical species or reactive radical species (RRS•), converting them in a long-live and less reactive radicals, mainly at the propagation and termination steps. Therefore, we propose a possible antioxidant mechanism for THC. This mechanism is showed in Equations (4) and (5):

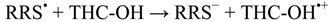
(4)

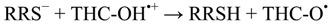
(5)


**Figure 6:**
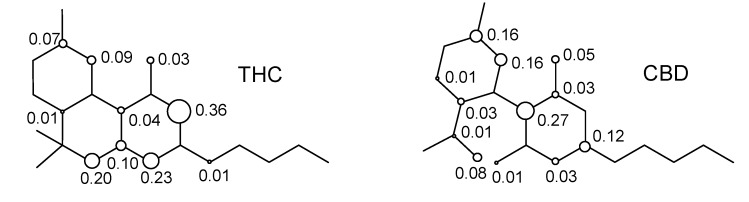
Spin densities in the cation free-radical of cannabinoid derivatives.

The resonance structures of the cation free-radicals obtained from the electron abstraction can be observed from the distribution of the spin densities for THC and CBD. Figure 6 shows the distribution of spin densities for the cation free-radicals of THC, as well as for the CBD compound.

From Figure 6, we can see that the calculated spin density for the initial electron abstraction of THC shows contributions of 0.20 for the oxygen of the ether moiety, 0.03 for the oxygen of the phenol group, 0.73 for the global contributions of the benzene ring, 0.17 for the cyclohexene ring, and 0.01 for the *n*-pentyl group. Other contributions are one order of magnitude smaller or show no contribution. Nevertheless, the CBD shows an increase of spin density contributions in the phenoxyl moiety of 0.05 and 0.01 for oxygen of the ether moiety, and reduction of 0.45 for the global spin contribution of the benzene ring. The limonene ring increases its spin contribution in 0.17 (THC) to 0.45 (CBD). Therefore, the lowest localization of the unpaired electron on the phenoxyl, ether, and alkyl groups, as well as the global spin contribution of the benzene ring, together with the localization of the unpaired electron on the double bonds, explain the highest stability of THC when compared to the CBD.

**Figure 7:**
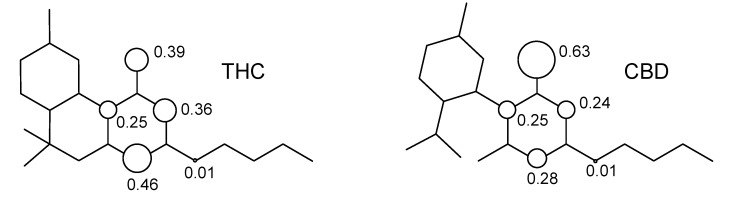
Structure of tetrahydrocannabinol (THC) and cannabidiol (CBD).

The resonance structures of the semiquinone free-radicals obtained from the hydrogen abstraction from the hydroxyl moiety can be observed from the distribution of spin densities for THC and CBD. Figure 7 shows the distribution of spin densities for the semiquinone free-radicals of these compounds.

We can see that the calculated spin density for the initial hydrogen abstraction of THC shows contributions of 0.39 for the oxygen of the phenol group, 0.25 and 0.36 for the carbon atoms at *ortho* positions, and 0.46 for the carbon atoms at *para* position of the benzene ring. The contributions of other atoms are almost one order of magnitude smaller or show no contribution. Nonetheless, the CBD shows an increase of spin density contributions in the phenoxyl moiety of 0.63 for oxygen of the phenol group, and reduction for the global spin contribution of the benzene ring of 0.25 and 0.24 for the carbon atoms at *ortho* positions, and 0.28 for the carbon atoms at the *para* position. Therefore, the highest localization of the unpaired electron on the phenoxyl group, as well as the decrease of the global spin contribution of benzene ring, explain the higher stability of THC when compared to the CBD molecule.

## 3. Computational Methodology

All calculations were performed with the Gaussian 09 molecular package [35]. Prior to any DFT calculations, a conformational search using the PM3 semiempirical method was applied [36]. In the geometry optimization was employed the B3LYP functional [37,38] with the 6–311++G(d,p) basis sets [39,40]. Frequency calculations were performed to confirm that the optimized structure is a true minima (no imaginary frequencies). Water solvation effects was included with the continuous surface charge polarizable continuum model IEF-PCM [41]. In addition, to better understand the antioxidant nature of the molecules studied here, we calculated the following properties: (i) energy of the highest occupied molecular orbital (HOMO); (ii) energy of the lowest unoccupied molecular orbital (LUMO); (iii) ionization potential (IP); (iv) hydroxyl bond dissociation energy (BDE_OH_); and (v) spin density. This methodology has been described in preview studies [42,43].

The adiabatic ionization potential (IP) was calculated as the energy difference between the neutral molecule and its respective cation free-radical (Equation 6). On the other hand, the semiquinone radical stability is usually calculated by the hydroxyl bond dissociation energies (BDE_OH_). The BDE_OH_ values were calculated as the energy difference between the neutral molecule and its respective semiquinone plus the hydrogen radical (Equation 7):

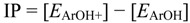
(6)

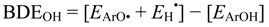
(7)


In the case of IP and BDEOH energies, an refinement of the electronic energies were performed by a single point energy with the M06-2X [44] functional employing the 6-311++G(3df,3pd) basis sets. The zero point vibrational energies (ZPVE) were obtained from the optimization with the B3LYP/6–311++G(d,p) method with the IEF-PCM continuum solvent model. Molecular electrostatic potential (MEP) surfaces were also obtained using Gaussian 09 [31], and the molecular visualization was performed with Molekel 4.2 [45].

## 4. Conclusions

The antioxidant mechanism of tetrahydrocannabinol (THC) and cannabidiol (CBD) was evaluated in this study. The results obtained indicate that these molecules have potential antioxidant properties in view of the fact their cation free-radicals show several resonance structures, in which the unpaired electrons are mainly distributed on the ether and alkyl moieties, as well as the benzene ring. These groups, along with the hydroxyl groups and double bonds, contribute to increase the HOMO value and to decrease the ionization potential (IP) and hydroxyl bond dissociation energies (BDE_OH_) values. The prevalent spin density contributions of these groups are determinant for the highest stability of the free-radicals due to more resonance structures. Another issue that may help in pointing out the antioxidant active of THC is related to the cyclohexenyl group at the *ortho* position of the phenol group when compared to the ether moiety at the *meta* position. Our calculated properties showed that THC molecules show a higher antioxidant potential than CBD. Therefore, we can conclude that our quantum chemical approach is a useful tool to determine the antioxidant ability of THC and its derivatives.
